# Examining the characteristic features of lipedema and the usefulness of BMI and WHtR in clinical evaluation

**DOI:** 10.1186/s12905-025-03834-9

**Published:** 2025-07-03

**Authors:** Monika Czerwińska, Marcin Gruszecki, Jacek Rumiński, Rita Hansdorfer-Korzon

**Affiliations:** 1 Department of Physiotherapy, Medical University of Gdańsk, 7 Dębinki Street, Gdańsk, 80-211 Poland; 2 Department of Radiology Informatics and Statistics, Faculty of Health Sciences, Medical University of Gdansk, Gdansk, Poland; 3 Department of Biomedical Engineering, Faculty of Electronics, Telecommunications and Informatics, Gdansk University of Technology, Gdansk, Poland

**Keywords:** Lipedema, Lipohyperplasia dolorosa, Lipoedema, BMI, WHtR, Quality of life

## Abstract

**Background:**

Lipedema is an adipose tissue disorder involving mostly women. One of the most characteristic lipedema symptoms is painful accumulation of adipose tissue in lower and upper extremities leading to disproportion. Due to the disproportionate body shape, it is recently thought that BMI (Body Mass Index) might not be fully sufficient to identify the weight ratios among lipedema patients and it is suggested to consider replacing BMI with WHtR (Waist-to-height ratio).

**Purpose:**

The aim of the study is to present the characteristic features of lipedema patients and the usefulness of BMI and WHtR among lipedema patients in reference to symptoms severity, quality of life and body composition.

**Methods:**

Forty-four women with lipedema were asked to rate their symptoms in a scale from 0 to 10, and to complete SF-36 questionnaire affecting quality of life. Participants also had body composition assessment.

**Results:**

Participants experienced various lipedema symptoms such as: heaviness in affected areas (97.7%), pain at palpation (100%), spontaneous pain (82%), disproportionate body shape and tendency to bruising (88.6%). The level of pain was strictly correlated with patients’ daily functioning (*R* = 0.79, *p* = 1.9*10^− 10^). The quality of life among participants measured with SF-36 was 57.4/100. WHtR enabled the same group of patients to be divided into three nearly equal groups, while BMI only divided them into two groups. Statistically significant differences could be observed both between BMI and WHtR groups.

**Conclusion:**

Lipedema symptoms have a direct impact on functioning of patients. Quality of life is decreased among women with lipedema. WHtR should be considered as a tool in identification of obesity among lipedema population.

**Supplementary Information:**

The online version contains supplementary material available at 10.1186/s12905-025-03834-9.

## Introduction

Lipedema is a chronic disorder of subcutaneous adipose tissue that primarily affects women [1–5]. Its onset is associated with hormonal changes during puberty, pregnancy or menopause [2, 6]. The disease has a genetic background and women with lipedema often report family history of this disorder in the female line [7, 8]. The exact genes responsible for the hereditary nature of lipedema are still unknown, however recent research has identified a possible gene connected to lipedema occurrence-AKR1C1 [8].

In recent years, lipedema has gained significant interest among the scientists and the general public, however, there are still many misconceptions and unanswered questions regarding this disease [3, 5, 9, 10]. Diagnosing lipedema remains challenging as a result of lack of specific diagnostic tests, and clear diagnostic criteria. Diagnosis is made upon clinical examination and it requires extensive knowledge of the characteristic signs of lipedema [2, 11]. Despite the increasing recognition of lipedema, awareness within the medical community is still insufficient [12, 13]. In many cases patients seek help for years, being misdiagnosed they are waiting for the correct diagnosis even for several years [9, 14–16]. Consequently, due to the high percentage of undiagnosed cases the accurate incidence is the unknown.

Lipedema is characterized by symmetric and bilateral pathological accumulation of adipose tissue due to adipocyte hypertrophy and hyperplasia [17, 18]. Although both hypertrophic and hyperplastic adipocytes are present in lipedema, only hypertrophic adipocytes are believed to be reducible through standard weight loss methods such as physical activity, diet, and bariatric surgery, while hyperplastic adipocytes can only be removed through liposuction [19].

Lipedema condition primarily affects the lower and/or upper extremities while sparing the trunk, hands, and feet, leading to a disproportionate body shape [1, 20]. Moreover women with lipedema experience pain (spontaneous or on palpation) in the affected areas, heaviness in the extremities, tendency to bruise easily [21, 22]. Recent German S2k Guidelines indicate that a tendency to bruise has not been scientifically proven to be associated with lipedema. Therefore, it should not be considered a specific symptom of lipedema, and used for diagnosing the condition [18]. Pain is thought to be the most characteristic sign of lipedema and its presence is required to make the diagnosis [23]. Some research suggest that the pain may be associated with inflammation in the affected area, which is also responsible for fibrosis of the tissue, however this hypothesis is not yet been confirmed [21]. The occurrence of inflammation in lipedema adipose tissue is thought to be connected with the presence of proinflammatory macrophages producing cytokines (interleukin-1β (IL-1β), IL-6, IL-12, IL-23, and TNF-α), increased sodium content, and interstitial fluid accumulation [11, 24, 25]. However, in a recent research by Wolf et al. the results revealed that lipedema is responsible for shifting the macrophages M2 to an immunosuppressive state, which impacts the inhibition of adipocyte progenitor proliferation, and as a result regulate glucose homeostasis [18, 26]. Although some studies suggest that swelling is not a characteristic feature of pure lipodema, and is usually connected with coexisting obesity [18], the presence and nature of fluid accumulation in affected patients remains under ongoing debate. A research by Allen, Herbst et al. showed that there is an evidence for lymphatic dysfunction in lipedema patients, possibly resulting from microangiopathy and impaired interstitial fluid clearance [24]. Additionally, a study by Pereira de Godoy et al. suggests a correlation between increasing body weight and the presence of edema in lipedema patients, indicating that obesity may significantly aggravate fluid retention in this population [27]. In contrast, a recent research by Mackie et al. showed that 85% of 44 lipedema patients presented normal lymphatic function during ICG lymphography evaluation. These findings highlight the need for further investigation to fully understand the involvement of lymphatics in lipedema population [28].

Lipedema is a medical condition, not merely an esthetic issue, and it can lead to disability [29]. The abnormal body shape and accumulation of adipose tissue often cause joint deformation, and may lead to loss of mobility [30]. All of the lipedema symptoms have a major impact on daily functioning and quality of life [31, 32].

The treatment of lipedema is focused mainly on pain reduction, relieving the symptoms, increasing functionality and quality of life [33]. Conservative treatment consists of the application of compression garments, physical activity, weight control and symptom reduction by using nutritional interventions such as anti-inflammatory, ketogenic or low-carbohydrate diets, and psychological support [18, 23, 33–37]. Surgical treatment, particularly liposuction, should also be considered, as it is currently the only method that can significantly reduce the volume and pain in the limbs affected by lipedema [38]. The importance of psychological support in lipedema patients is emphasized by scientists, since women with lipedema struggle with eating disorders, depression, and other mental health problems such as hopelessness [32]. 

Lipedema patients often additionally struggle with obesity [23, 39]. Various studies show that more than 50% of lipedema women are also obese [9, 14, 31, 40, 41]. Recent German guidelines indicate that lipedema does not lead to obesity, and obesity in lipedema patients is not a result of the lipedema itself [18]. These two conditions should be considered distinct, although they often coexist [42]. Therefore, accurately measuring obesity in lipedema patients presents a challenge. The indicator that is typically used to determine overweight and obesity is Body Mass Index (BMI). However, recent research indicates that BMI may not be the most accurate measure of obesity in lipedema patients, as it focuses solely on total body mass without accounting for the disproportionate body shape seen in these individuals [43]. Due to disproportionately low visceral fat and high peripheral fat accumulation among lipedema patients, BMI often overestimates metabolic risk in this group of patients. Therefore, alternative measurements should be considered for assessing obesity in lipedema patient [43]. Researchers suggest using the waist-to-height ratio (WHtR) instead, as it considers both waist circumference and height, and is independent of total body weight, making it a more accurate measure for obesity in lipedema [42–44].

The purpose of the study is to present the characteristic features of lipedema, the level of quality of life, the severity of lipedema symptoms, and body composition among lipedema patients, based on BMI and WHtR.

## Materials and methods

### Participants

**Fig. 1:**
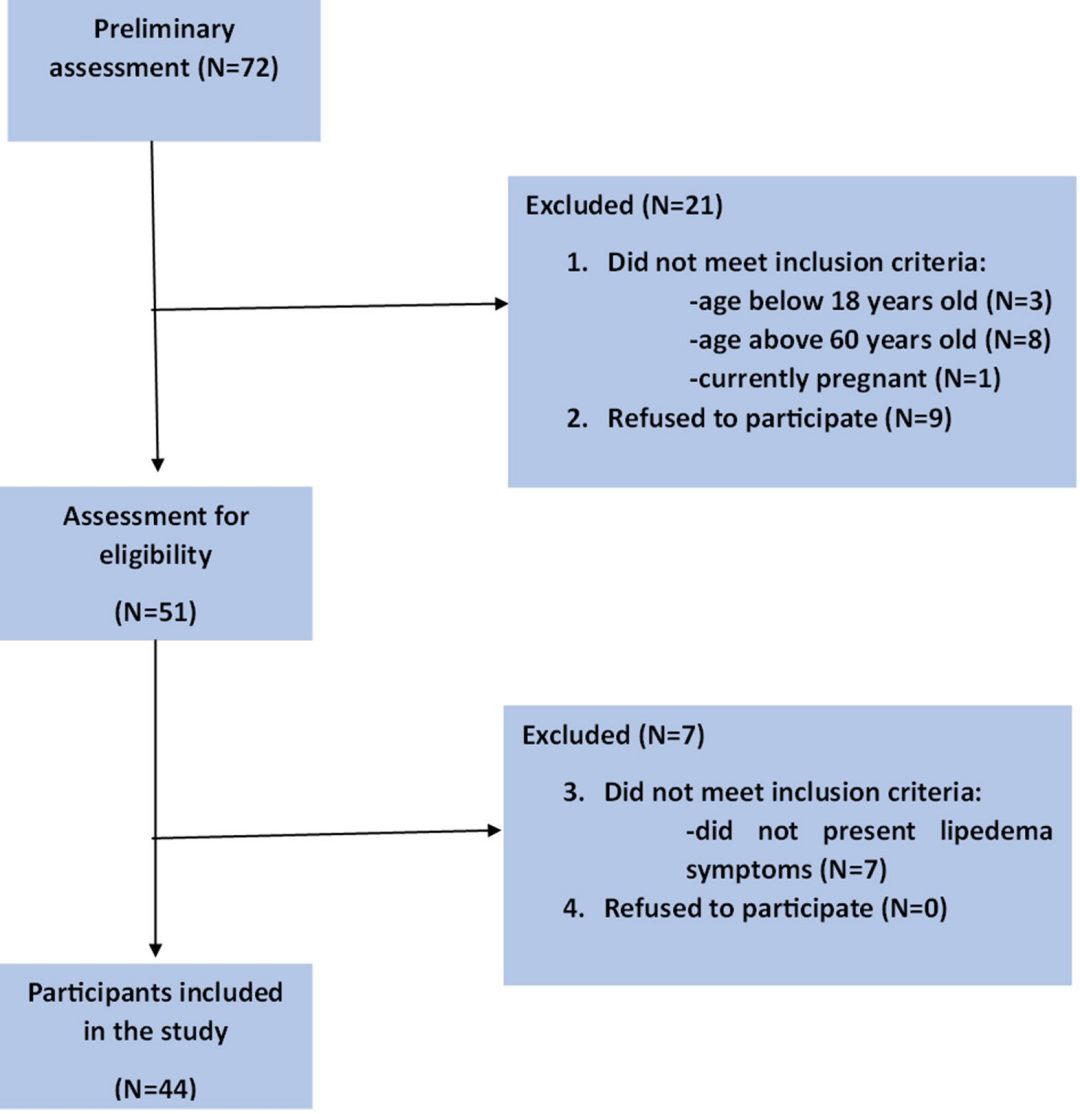
Flow chart presenting the study qualification process

Patients were recruited from outpatient clinics in Poland and social media groups for women struggling with weight disorders. The study received the ethical consent from Medical University of Gdansk Ethical Committee (Approval number-912/2021–2022) in accordance with the Declaration of Helsinki. The recruitment of participants took part from April 2023 until September 2023. Those meeting the primary inclusion criteria (female, age between 18 and 60, currently not pregnant) were invited to further qualification concentrated on identification of women with lipedema symptoms. The assessment consisted of medical history regarding lipedema characteristic features, visual evaluation of adipose tissue deposition, palpation to assess tissue consistency and sensitivity, as well as Stemmer sign and pitting tests. Women with lipedema symptoms were included in the study. The exclusion criteria were: male sex, age below 18 and above 60, lack of lipedema symptoms, pregnancy. The flow chart presents the process of qualification of participants (Fig. 1).

### Assessment methods

All of the qualified women had evaluation of quality of life, severity of lipedema symptoms, and measurement of height, weight, waist and hip circumference, and body composition. The evaluation was conducted by a qualified-lymphological physiotherapist. Participants completed paper versions of the SF-36 and symptom severity survey questionnaires onsite, with the data subsequently digitized into a database. Height, waist, and hip circumference measurements were taken by a qualified physiotherapist using a measuring tape.

Quality of life was measured with SF-36 questionnaire in eight dimensions: Physical functioning, Role limitations due to physical health, Role limitations due to emotional problems, Energy/fatigue, Emotional well-being, Social functioning, Pain, and General health. The rating scale ranged from 0 (worst) to 100 (best) and applied to all of the above mentioned dimensions. The Polish version of SF-36 questionnaire was used in the study [45].

The severity of lipedema symptoms was assessed by a survey where participants rated the subjective intensity of each specific feature on a 0–10 scale. A similar scale was used in our previous study [46]. A score of 0 indicated the absence of the feature, while 10 indicated the highest possible intensity. Items that were included in the survey were: Spontaneous Pain (Visual Analogue Scale), Subjective level of disproportion between trunk and hips, Subjective level of swelling in lower limbs, Subjective level of heaviness in extremities, Subjective level of intensity of bruising, Subjective level of sensitivity to touch, Subjective level of accumulation of fatty tissue around hips/thighs, The impact of lipedema symptoms on daily activities.

The evaluation of weight and body composition (Percentage of fluid, Total percentage of fat, Trunk % fat tissue, Right leg %of fat, Left leg %of fat, Muscle mass(kg), Trunk Muscle mass, Right leg Muscle mass, Left leg Muscle mass were performed using Tanita BC-545 N with bioelectrical impedance. Participants were required to wear either underwear or a swimsuit during the analysis.

### Statistics

To estimate the correlation between features collected during the study, the Pearson correlation coefficient was calculated and corresponding p-values. To fit a straight line through the points, the least squares method was employed. The results of our calculations are presented in Fig. 1.

**Fig. 2:**
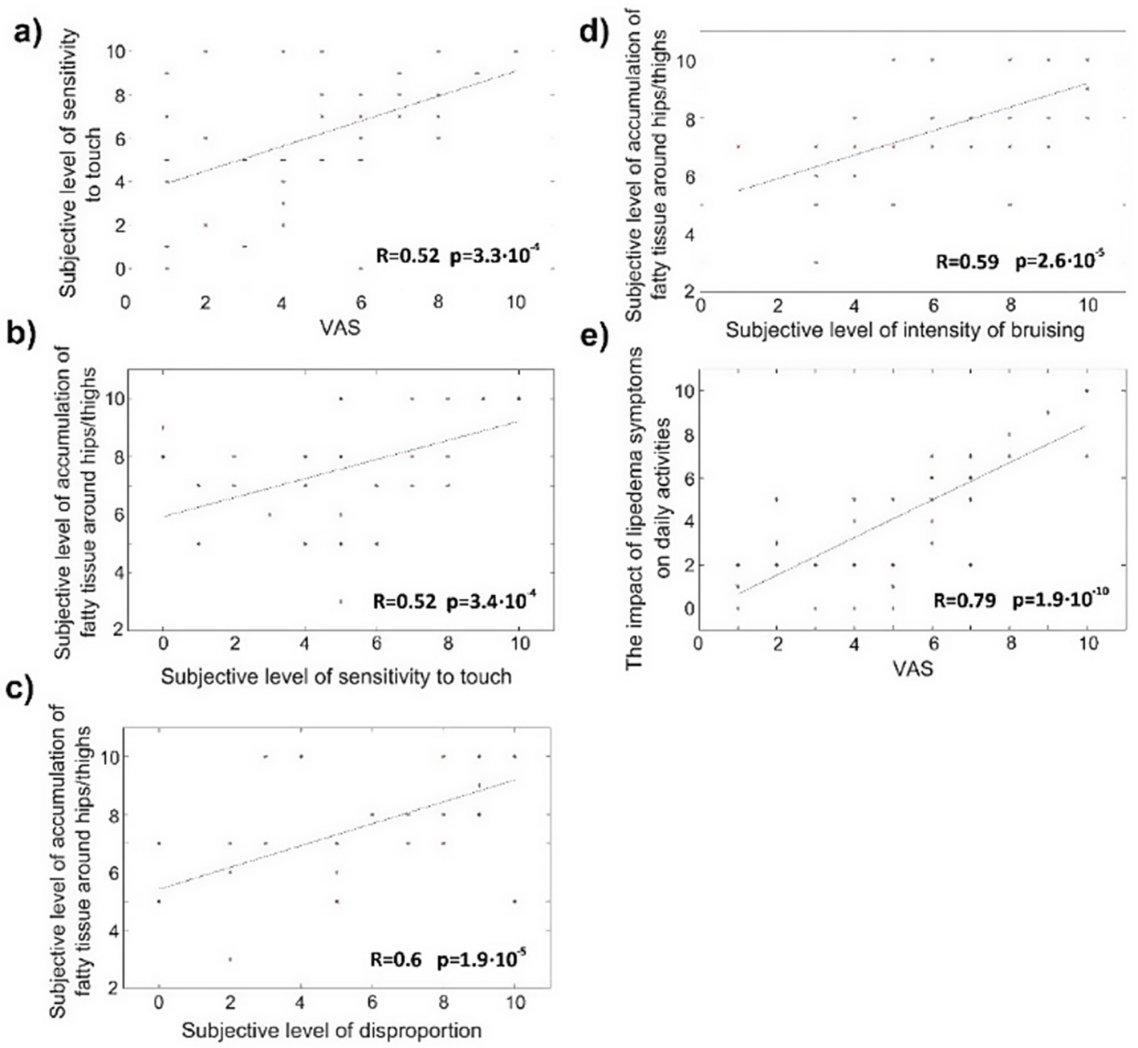
Scatter plots illustrate the relationship between data collected during the experiment. A straight line was fitted to the data using the least squares method, and Pearson’s correlation coefficient is displayed in the bottom right corner of the plot

Nonparametric statistical tests were employed for all comparisons to avoid assuming normality in the results. The Mann-Whitney U test was used to assess whether the median of two BMI groups (below and above 30) differed significantly. The results of these calculations are shown in Fig. 2.

**Fig. 3:**
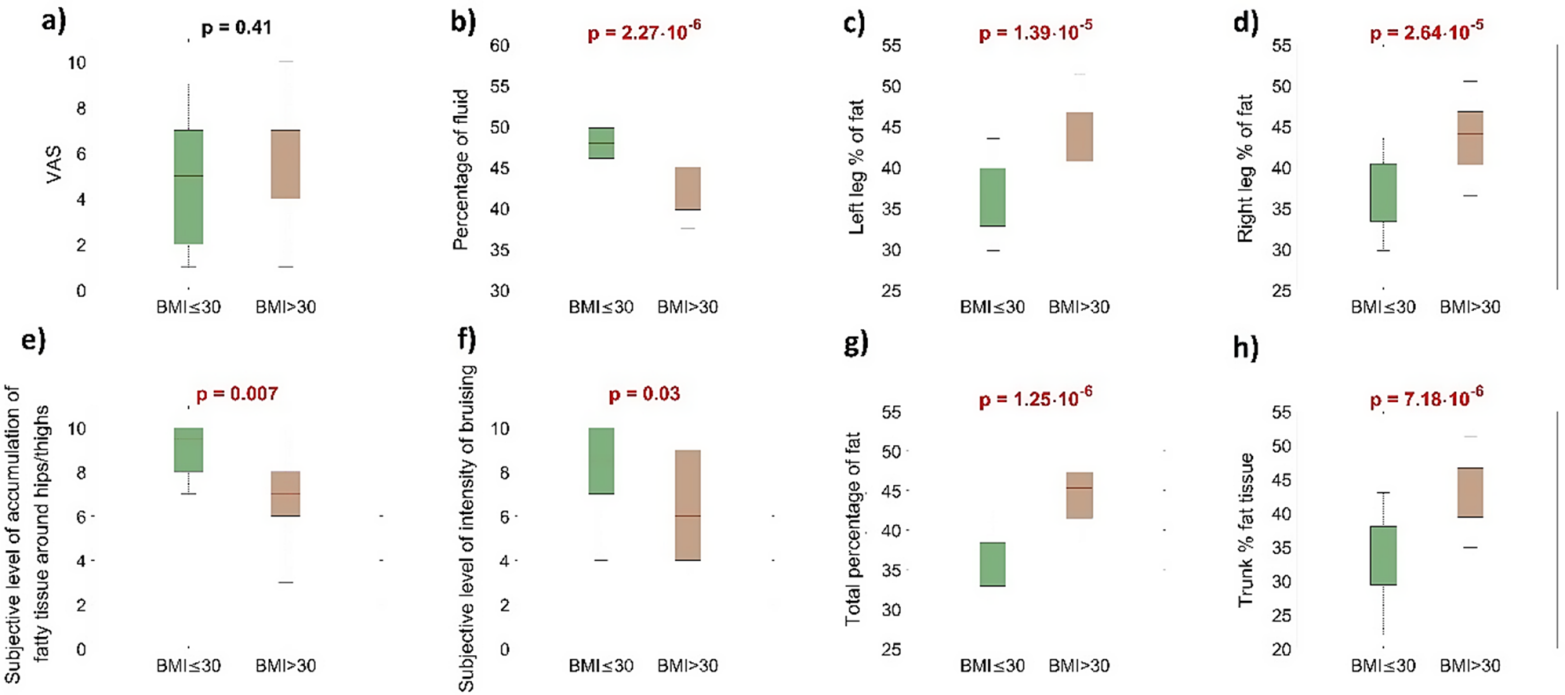
Boxplots illustrate the results of the analysis for the collected data. Two box colours correspond to two groups of volunteers with ‘BMI ≤ 30’ and ‘BMI > 30’. The ‘p’ values were estimated using the Mann-Whitney U Test

Furthermore, we investigated the effects related to the WHtR parameter. We categorized volunteers into three groups: normal, obese, and overweight. For all comparisons among these three groups of volunteers, a non-parametric Kruskal-Wallis test was conducted to identify differences. Post hoc comparisons, using the Dunn test, were performed to determine specific differences between groups of volunteers when the Kruskal-Wallis test was statistically significant (*p* < 0.05). The results of these analyses are presented in Fig. 3.

## Results

### Patient characteristics

A total number of 44 met the study’s inclusion criteria. All participants were categorized into groups based on Body Mass Index and Waist-to-Height Ratio. For the purposes of the analyses in this study the division depending on BMI was made by assigning women into two groups (BMI below 30, and BMI above 30), since there were only three women with ‘normal body weight’ according to this parameter. The number of participants in the group with BMI below 30 kg/m^2^ was 18, and in the group with BMI above 30 kg/m^2^ was 26. Waist-to-height ratio is calculated using waist circumference and height. Normal weight was established with WHtR 0.40–0.50, overweight with WHtR 0.51–0.56, and obesity above 0.57 for women up to 40 years old. For participants older than 40 years old WHtR increased 0.01 per year, and for women above 50 years old normal weight was determined with WHtR 0.5–0.6, overweight 0.61–0.66 and obesity with WHtR above 0.67 [43]. The allocation to the groups depending on WHtR was based on the data included in the recent study regarding the use of this parameter in lipedema patients. Participants were divided into 3 groups ‘normal’-15 participants, ‘overweight’-13 participants and ‘obesity’-16 participants. Table 1 presents the characteristics of participants included in the study.

**Table 1:** The characteristics of patients included in the study

Feature	Total (*n* = 44)
Age, years (median$\:\frac{Q3}{Q1}$)	$\:{38}_{31.75}^{44.25}$
BMI	
Normal	3 (6.8%)
Overweight	15 (34%)
Obesity	26 (59.2%)
WHtR	
Normal	15 (34%)
Overweight	13 (29.5%)
Obesity	16 (36.5%)
Body weight (kg)	89.6
Previous lipedema diagnosis	
Yes	2 (4.5%)
No	42 (95.5%)
Lipedema symptoms	
Heaviness in lower extremities	43 (97.7%)
Pain at palpation	44 (100%)
Spontaneous pain in lower/upper extremities	36 (81.8%)
Disproportion between slimmer trunk and enlarged limbs	39 (88.6%)
Easy bruising	39 (88.6%)
Accumulation of fat tissue mostly around legs	40 (90.9%)
Difficulties losing weight in the affected areas	42 (95.45%)
Swelling around the ankles depending on temperature	36 (81.8%)
Lipedema Stage	
Stage 1	14 (31.8%)
Stage 2	22 (50%)
Stage 3	8 (18.2%)
Family history	
Yes	36 (81.8%)
No	8 (18.2%)
Onset	
Puberty	37 (84%)
Pregnancy	4 (9%)
Menopause	1 (2%)
Other	2 (5%)
Declared level of physical activity	
None	7 (15.9%)
Low	13 (29.5%)
Medium	19 (43%)
High	5 (11.6%)

### Lipedema symptoms

All Participants were asked to rate the severity of each symptom in 0–10 scale. The correlations between lipedema symptoms among women were assessed. Figure 2 displays five scatter plots illustrating the relationships between various parameters gathered during the experiment. Correlation coefficients R were calculated for the assembled parameters, and all estimated R values were found to be statistically significant (*p* < 0.05). The analysis revealed four instances of moderate correlation (R ∈ [0.5, 0.7]): Subjective level of sensitivity to touch vs. pain (VAS), (Fig. 2a); Subjective level of accumulation of fatty tissue around hips/thighs vs. Subjective level of sensitivity to touch, (Fig. 2b); Subjective level of accumulation of fatty tissue around hips/thighs vs. Subjective level of disproportion, and (Fig. 2c); Subjective level of accumulation of fatty tissue around hips/thighs vs. Subjective level of intensity of bruising. (Fig. 2d)

Additionally, one correlation was identified as high (R ∈ [0.7, 0.9]): the impact of lipedema symptoms on daily activities vs. pain (VAS).(Fig. 2e).

### Quality of life, symptom’s severity and body composition depending on BMI

The level of quality of life by SF-36 form and symptom’s severity were assessed among all of the participants. The results of quality of life and symptom’s severity depending on BMI are presented in Table 2.

**Table 2:** Symptoms severity and quality of life depending on BMI (BMI^1^-below 30 kg/m^2^, BMI^2^-above 30 kg/m^2^), WHtR ^1^-normal weight, WHtR^2^-owerweight, WHtR^3^ obesity; median values, first quartile (lower index) and third quartile (upper index) are presented in the table

Feature	Total	BMI^1^ (*n* = 18)	BMI^2^ (*n* = 26)	BMI pValue	WHtR^1^ (*n* = 15)	WHtR^2^ (*n* = 13)	WHtR^3^ (*n* = 16)	WHtR *p* Value
VAS (0–10)	$\:{5.5}_{3.75}^{7}$	$\:{5}_{2}^{7}$	$\:{6}_{4}^{7}$	0.40	$\:{6}_{2.5}^{7}$	$\:{5}_{3.75}^{6.25}$	$\:{6}_{74}^{7}$	0.84
Subjective level of disproportion (0–10)	$\:{8}_{5}^{9}$	$\:{8}_{6.25}^{9}$	$\:{7}_{5}^{8.75}$	0.38	$\:{8}_{6.5}^{8.5}$	$\:{6.5}_{5}^{10}$	$\:{8}_{5}^{8}$	0.94
Subjective level of sensitivity to touch (0–10)	$\:{7}_{5}^{8}$	$\:{7.5}_{5}^{8.75}$	$\:{7}_{5}^{8}$	0.57	$\:{7}_{4.5}^{8}$	$\:{7.5}_{6.5}^{9}$	$\:{6}_{3}^{8}$	0.23
Subjective level of heaviness in extremities (0–10)	$\:{7}_{5}^{9.25}$	$\:{7}_{4.25}^{8.75}$	$\:{7}_{5}^{9.25}$	0.56	$\:{7}_{4}^{8.5}$	$\:{8}_{5}^{9.25}$	$\:{6}_{4}^{10}$	0.5
Subjective level of intensity of bruising	$\:{7.5}_{5}^{10}$	$\:{8.5}_{7}^{10}$	$\:{6}_{4.25}^{8.75}$	**0.03**	$\:{8}_{7}^{10}$	$\:{7.5}_{6}^{10}$	$\:{5}_{3}^{8}$	**0.026**
Subjective level of swelling in lower limbs (0–10)	$\:{7}_{5.75}^{9.25}$	$\:{7}_{6.25}^{8.75}$	$\:{7.5}_{5.25}^{9.75}$	0.99	$\:{7}_{5.5}^{8.5}$	$\:{8}_{7}^{10}$	$\:{6}_{4}^{8}$	0.27
Subjective level of accumulation of fatty tissue around hips/thighs (0–10)	$\:{8}_{7}^{10}$	$\:{9.5}_{8}^{10}$	$\:{7}_{6.25}^{8}$	**0.007**	$\:{8}_{7.5}^{10}$	$\:{8}_{7}^{10}$	$\:{7}_{5}^{8}$	0.1
Subjective impact on daily activities (0–10)	$\:{5}_{2}^{7}$	$\:{2.5}_{1.25}^{5}$	$\:{5}_{2.25}^{7}$	0.09	$\:{2}_{1.5}^{6}$	$\:{5}_{2.75}^{7}$	$\:{5}_{2}^{7}$	0.56
SF-36 Total (0-100)	$\:{57.4}_{45.6}^{69.6}$	$\:{55.62}_{45}^{68.9}$	$\:{61.87}_{49.86}^{69.9}$	0.47	$\:{55.97}_{43.2}^{68.9}$	$\:{63.5}_{53.3}^{69.6}$	$\:{53.88}_{43}^{70}$	0.37
SF-36 Physical Functioning (0-100)	$\:{80}_{65}^{90}$	$\:{82.5}_{71.3}^{90}$	$\:{75}_{65}^{85}$	0.21	$\:{85}_{75}^{90}$	$\:{75}_{68.8}^{86.3}$	$\:{75}_{45}^{85}$	0.26
SF-36 Role limitations due to physical health (0-100)	$\:{75}_{43.8}^{100}$	$\:{50}_{6.25}^{100}$	$\:{75}_{75}^{100}$	0.09	$\:{50}_{12.5}^{100}$	$\:{66.68}_{33.33}^{100}$	$\:{100}_{33.33}^{100}$	0.41
SF-36 Role limitations due to emotional health (0-100)	$\:{66.6}_{0}^{100}$	$\:{33.33}_{0}^{100}$	$\:{66.6}_{33.33}^{100}$	0.1	$\:{33.33}_{0}^{83.3}$	$\:{66.68}_{33.33}^{100}$	$\:{100}_{33.33}^{100}$	0.15
Energy/fatigue (0-100)	$\:{45}_{33.75}^{60}$	$\:{40}_{31.25}^{60}$	$\:{45}_{35}^{58.75}$	0.5	$\:{40}_{25}^{55}$	$\:{55}_{40}^{60}$	$\:{45}_{35}^{50}$	0.35
Emotional well-being (0-100)	$\:{60}_{40}^{72}$	$\:{58}_{38}^{74}$	$\:{60}_{42}^{72}$	0.9	$\:{52}_{36}^{68}$	$\:{64}_{52}^{72.75}$	$\:{60}_{40}^{64}$	0.27
Social functioning (0-100)	$\:{50}_{50}^{75}$	$\:{50}_{40.6}^{75}$	$\:{62.5}_{50}^{75}$	0.4	$\:{50}_{43.75}^{68.75}$	$\:{62.5}_{50}^{78.12}$	$\:{50}_{50}^{75}$	0.56
Pain (0-100)	$\:{47.5}_{32.5}^{78.12}$	$\:{48.75}_{15}^{80}$	$\:{47.5}_{35}^{69.375}$	0.85	$\:{47.5}_{12.5}^{73.75}$	$\:{55}_{36.8}^{80}$	$\:{47.5}_{25}^{67.5}$	0.3
General health (0-100)	$\:{35}_{20}^{50}$	$\:{40}_{27.5}^{50}$	$\:{35}_{20}^{48.75}$	0.37	$\:{40}_{27.5}^{50}$	$\:{40}_{23.75}^{61.25}$	$\:{25}_{15}^{40}$	0.37

The level of pain among all of the participants was rated or 5.5/10, the level of disproportion between trunk and hips for 8/10, heaviness in extremities for 7/10, sensitivity to touch, bruising, level of swelling, and accumulation of adipose tissue mostly around hips/thighs was rated for 7/10, 7.5/10, 7/10 and 8/10, respectively. Participants valued the impact of lipedema symptoms on daily activities for 5/10. The comparison of groups depending on BMI (below 30 and above 30) revealed statistically significant differences in 2 symptoms- intensity of bruising and accumulation of tissue around hips/thighs. (Fig. 2e, f) The differences in remaining symptoms were not significant.

Total SF-36 among all of the participants was 57.4/100. The quality of life in specific dimensions was 80, 75, 66.6, 45, 60, 50, 47.5, 35 for Physical Functioning, Role limitations due to physical health, Role limitations due to emotional health, Energy/fatigue, Emotional well-being, Social functioning, Pain and General health respectively. Statistically significant differences between BMI groups could not be found.

The results of the Mann-Whitney U Test are presented in Fig. 3.

We observed seven statistically significant differences (*p* < 0.05) between the two BMI groups of volunteers for the specified parameters. However, no statistically significant difference (*p* > 0.05) was found for the level of pain (VAS), which suggests that the level of pain is not connected to BMI (Fig. 3a). Women with higher BMI had significantly lower percentage of tissue fluid, higher percentage of fat content in general, in the left and right lower extremity and trunk area.

### Quality of life, symptom’s severity and body composition depending on WHtR

**Fig. 4:**
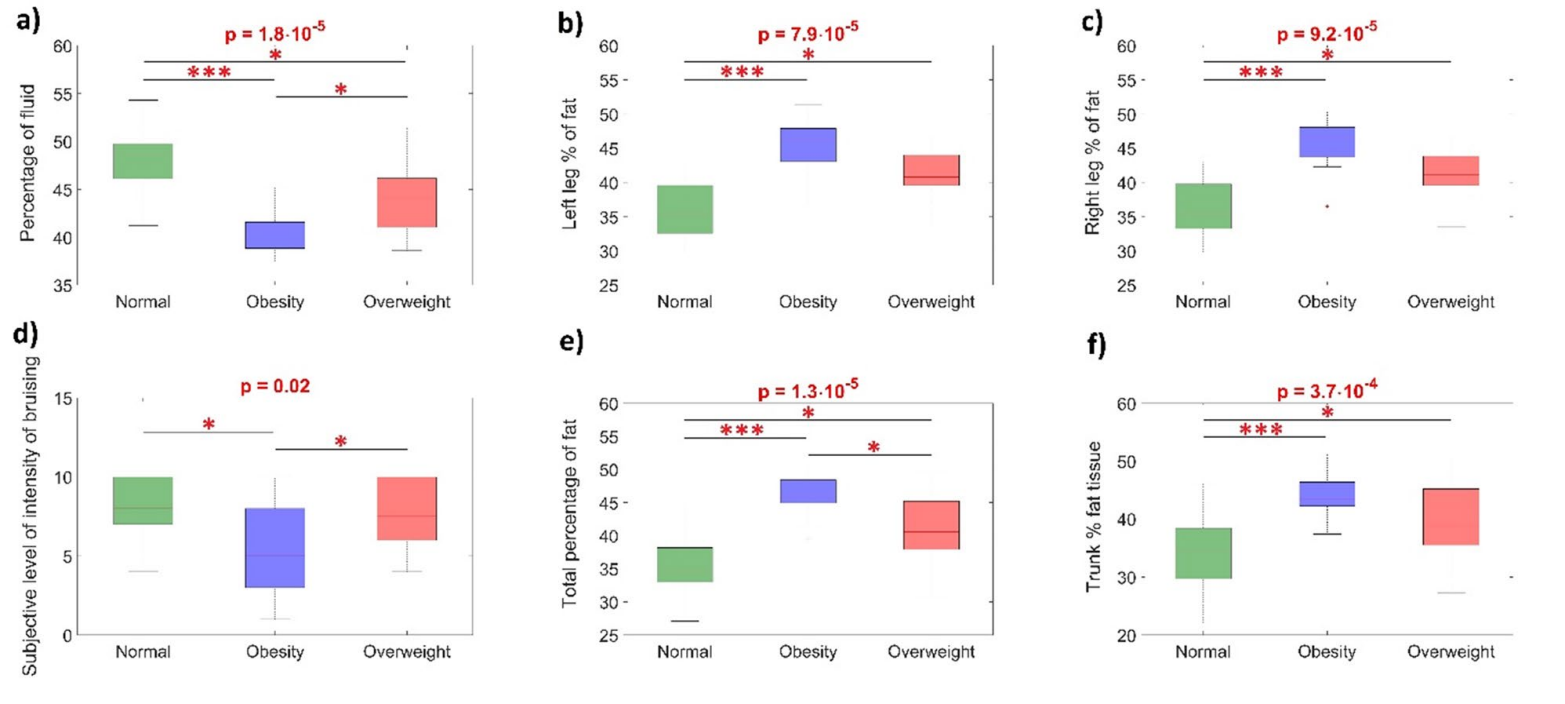
The results of the statistical analysis for the collected data are presented. The ‘p’ values were estimated using the Kruskal-Wallis test. Post hoc test comparison (Dunn test) was employed to identify differences among three groups of volunteers concerning the WHtR parameter (‘Normal,’ ‘Obesity,’ and ‘Overweight’ groups). *p < 0.05; **p < 0.01; ***p < 0.001

The differences in quality of life and symptom severity in WHtR groups are presented in Table 2. Statistically significant difference was observed in intensity of bruising. (Fig. 4d) The remaining parameters remained statistically unsignificant.

To compare the WHtR parameter among three groups, the Kruskal-Wallis test was employed. The results of the Kruskal-Wallis test were found to be statistically significant for all considered parameters (Fig. 4). Furthermore, employing post hoc Dunn test revealed statistically significant differences (*p* < 0.05) among the three groups with respect to the WHtR parameter.

## Discussion

Lipedema is a complex condition of adipose tissue affecting women. Despite growing awareness, the disease remains poorly recognized, and misconceptions hinder proper diagnosis and proper treatment [12, 13]. Women with lipedema usually struggle with painful symptoms, and mental health issues that have a direct impact on their daily functioning [23]. Some research suggests that psychological distress can have an impact on lipedema symptoms [18, 23, 40, 47]. However, recent research by Dinnendahl et al. showed that pain experienced by lipedema patients, measured with validated quantitative sensory testing is rather somatic not psychosomatic, and lipedema patients did not present any psychological abnormalities [48]. All participants in our study reported experiencing pain upon palpation, and 97.7% reported heaviness in the affected areas. Spontaneous pain, disproportion between the trunk and extremities, and easy bruising were present in 82% and 88.6% of participants, respectively. Even though a tendency to bruise has not been proven to be a specific feature of lipedema, many patients experience this condition. Moreover, participants with higher level of accumulation of adipose tissue mainly around hips/thighs reported statistically higher levels of disproportion, bruising and sensitivity to touch. Higher level of pain (VAS) was connected to higher sensitivity on palpation. The observed correlation between the intensity of bruising and the level of adipose tissue accumulation in the hips and thighs may suggest that specific lipedema-related adipose tissue distribution could contribute to increased bruising tendency reported by patients in affected areas. While many lipedema patients report a heightened susceptibility to bruising, further research is needed to better understand and confirm this relationship.

Quality of life in lipedema patients was previously assessed by Romejn [14]. The study included 163 lipedema patients and evaluated the quality of life using RAND-36 questionnaire, which is similar to SF-36 and measures the quality of life in nine health related dimensions. The total RAND score was 59.3/100, physical functioning was rated for 63.5, social functioning was 67.3, emotional limitations score was 71.6, mental health, vitality, pain general health and health change were rated for 69.9, 49.1, 57.2, 51.6, and 51.4 respectively [14]. In our study the medial value of total SF-36 score was 57.3, the values of physical functioning, role limitations due to physical health, role limitations due to emotional health, energy/fatigue, emotional well-being, social functioning, pain and general health were valued for 80, 75, 66.6, 45, 60, 50, 47.5, and 35 respectively.

A different study explored the quality of life using SF-36 in Polish healthy population (3,511 participants) [49]. The medial value of total SF-36 was 61.6, and the values of physical functioning, role limitations due to physical health, role limitations due to emotional health, vitality, mental health, social functioning, bodily pain and general health were valued for 95, 75, 100, 60, 46.7, 75, 67.5, and 55 respectively [49]. The comparison of the above mentioned data suggest that women with lipedema have decreased quality of life in contrast to the general population.

Pain levels in lipedema patients by visual analog scale have been described by various researchers. The level of pain in a study by Forner-Cordero was 5.7/10 among 138 lipedema patients [44]. Our study indicated that higher pain levels correlate with more difficulties in daily activities. Moreover, the level of experiencing pain among participants in our study was rated for 5.5/10 and is was not correlated with either BMI or WHtR.

Lipedema symptoms severity was previously assessed by Dudek. The study evaluated the intensity of symptoms on a scale from 0 to 5. The level of heaviness in legs, fatigue, swelling, fat tissue pain, muscle pain and itching were rated for 3.36/5, 3.74/5, 3.63/5, 3.16/5, 2.9/5 and 2.49 respectively [31]. In our study heaviness in affected areas, swelling and pain at palpation were rated for 7/10, which presents similar results as the above mentioned study.

Body Mass Index is widely used to identify the metabolic risk, by categorizing individuals based on body weight and height. In lipedema one of the most characteristic features is disproportion between trunk and the hips/limbs. The usage of BMI may not be a fully appropriate tool to identify the metabolic risk since it does not consider the disproportionate body shape in lipedema. Moreover Body Mass Index may be increased in lipedema patients without existence of obesity [20, 43]. Recent studies found that despite increased BMI women with lipedema have a lower risk of diabetes than women without lipedema in the same BMI range [35, 50]. In a study involving 160 lipedema patients with average BMI 39 kg/m^2^ only 0.2% had diabetes [51]. In a different study of 209 women with lipedema only 1% had diabetes type 2, 1.4% diabetes type 1 and 7.2% had dyslipidemia [15]. Another study showed that women with lipedema (*n* = 13) exhibit superior glucose metabolism, as indicated by their lower HbA1c levels compared to the control group (*n* = 13) [52].

Another parameter used to identify metabolic risk is WHtR. It is thought to be more specific for lipedema patients since it uses waist circumference and height, and may have an outlook for disproportionate adipose tissue deposition in lipedema [43]. According to a study by Forner-Cordero out of 138 lipedema patients 23.2% had normal body weight, 31.2% were overweight, 37.7% had obesity Class 1 and 8% obesity class 2 using BMI, while using WHtR the same participants were slim (10.1%), healthy (31.1%), overweight (19.6%), very overweight (15.9%) and obese (23.2%) [44]. In another study of 607 lipedema patients 18.5% were normal weight, 30.3% were overweight and 51.2% had obesity calculated using BMI, while based on WHtR 15.2% of patients were underweight, 45.5% normal weight, 22.1% overweight and 17.3% obese [43]. 

In our study there were statistically significant differences regarding lipedema symptoms and body composition both in WHtR and BMI groups. However, considering that WHtR allowed us to categorize the same cohort into three nearly equal groups, whereas BMI only separated them into two, and in the light of recent studies indicating that women with lipedema may have a reduced risk of lifestyle diseases, such as diabetes, compared to non-lipedema patients matched by BMI, WHtR should be regarded as a more precise tool for assessing lipedema patients. It facilitates the accurate identification of whether a lipedema patient has co-existing obesity, which is generally linked to an elevated risk of lifestyle diseases, or whether the patient has been erroneously classified as obese based on BMI alone, despite having a disproportionate body shape and a potentially lower risk of such diseases.

Another tool used among lipedema patients is the waist-to-hip ratio (WHR) [53]. While WHR is useful for assessing the degree of disproportion between the trunk and hips, it may underestimate obesity in cases of significant disproportion, potentially presenting a normal value when the patient should be classified as obese [54, 55]. Furthermore, changes in this ratio depend on both hip and waist circumference, meaning it can decrease either with an increase in hip circumference or a decrease in waist circumference [55].This limitation highlights the advantage of WHtR over WHR.

Body composition in relation to BMI, measured by DXA (dual-energy X-ray absorptiometry), was previously assessed among lipedema patients by Dietzel et al. The study found that women with lipedema within the normal BMI range had a leg fat mass adjusted for BMI at 0.53 and a trunk fat mass at 0.42. In the overweight group, the leg fat mass was 0.55 and the trunk fat mass was 0.57. In the obesity group, the leg fat mass was 0.6 and the trunk fat mass was 0.66 [56]. These results indicate that both trunk and leg fat increase with rising BMI. In our study, body fat was measured as a percentage of body weight, and the results show a significant increase in both leg fat percentage and trunk fat percentage in women with higher BMI. While WHtR (waist-to-height ratio) has not previously been correlated with body composition measurement among lipedema patients, our study found that body fat percentage also increases with WHtR.

Our study revealed a correlation between experiencing pain and pain of fatty tissue on palpation. Moreover, a higher level of accumulation of adipose tissue mostly around hips/legs was correlated with a higher intensity of bruising and pain on palpation. The level of experiencing pain was connected with a higher impact of lipedema symptoms on daily functioning. A study conducted by Dudek showed that lower quality of life among lipedema patients is correlated with higher severity of lipedema symptoms [47].

The main limitation of this study is that it was based on single observation, and did not present any changes in time. Moreover, it would be useful in future research to focus more on assessment of quality of life, and lipedema symptoms in longer periods of time, and on comparing the usage of BMI and WHtR as a tool for assessing the effectiveness of lipedema treatment. Moreover, our study primarily focused on the lower extremities, as they are the region most commonly affected by lipedema. However, we believe that future research should also provide a more detailed examination of ailments in the upper extremities.

## Conclusions

Women with lipedema experience various symptoms that have direct impact on their daily functioning. Lipedema patients have decreased quality of life compared to non-lipedema population. Women with lipedema have a lower diabetes prevalence than BMI matched controls. The results of our study suggest that WHtR should be considered as a more accurate tool in identifying metabolic risk in lipedema patients and should be used widely both in lipedema research and in clinical setting. Furthermore, WHtR could be a valuable tool for monitoring the effects of therapeutic interventions.

## Electronic supplementary material

Below is the link to the electronic supplementary material.

Supplementary Material 1

Supplementary Material 2

## Data Availability

Data presented in this study is available on request from the corresponding author, due to the confidential patient information included in database.
