# Supplementary material for: Examining the characteristic features of lipedema and the usefulness of BMI and WHtR in clinical evaluation
Source: BMC Womens Health. 2025 Jul 3;25:292. doi: 10.1186/s12905-025-03834-9 (PMC12225527; doi:10.1186/s12905-025-03834-9)
Supplement: Supplementary file 1 — Supplementary Material 1 [file 12905_2025_3834_MOESM1_ESM.docx]

**Assessment of Lipedema Symptom Severity**

1. On a scale of 0 to 10, how would you rate the severity of pain you experience in areas affected by lipedema? Please write down your answer. (0 – no pain, 10 – maximum possible level of pain):
   ......................................................................................................................................................
2. Please indicate the extent to which the following symptoms affect you on a scale of 0-10. 0 – symptom is absent, 10 – symptom is severe and causes significant discomfort:

- Disproportion between a slimmer torso/upper body and extremities:
  ..................................................................................................................................................
- Feeling of leg swelling increasing during the day:
  ......................................................................................................................................................
- Feeling of heaviness in the legs:
  ......................................................................................................................................................
- High tendency to bruise:
  ......................................................................................................................................................
- Pain in the extremities when touched/pressed:
  ......................................................................................................................................................
- Accumulation of fat tissue primarily in the legs/hips/thighs:
  ......................................................................................................................................................

1. Please indicate the extent to which the above symptoms affect your ability to perform daily activities (on a scale of 0 to 10, where 0 means no pain and 10 means unbearable pain):
   ......................................................................................................................................................
